# Crossing the perspectives of patients, families, and physicians on cancer treatment: A qualitative study

**DOI:** 10.18632/oncotarget.12770

**Published:** 2016-10-19

**Authors:** Massimiliano Orri, Jordan Sibeoni, Guilhem Bousquet, Mathilde Labey, Juliette Gueguen, Cyril Laporte, Sabine Winterman, Camille Picard, Clara Nascimbeni, Laurence Verneuil, Anne Revah-Levy

**Affiliations:** ^1^ Service Universitaire de Psychiatrie de lAdolescent, Argenteuil Hospital Centre, Argenteuil, France; ^2^ ECSTRA Team, UMR-1153, Inserm, Paris Diderot University, Sorbonne Paris Cite, Paris, France; ^3^ Université Paris Diderot, Sorbonne Paris Cité, Laboratoire de Pathologie, UMR-S 1165, Paris, France; ^4^ INSERM, U1165-Paris, France; ^5^ Université Paris 1, Léonard de Vinci, Villetaneuse, France; ^6^ AP-HP-Hôpital Avicenne, Service d’Oncologie Médicale–Bobigny, Bobigny, France; ^7^ Ecole Doctorale E420, Paris Saclay University, Paris, France; ^8^ Centre de Cancérologie Paris Nord, Sarcelles, France; ^9^ Recherche Innovation Santé Sarcelles, Sarcelles, France; ^10^ Department of Dermatology, CHRU Caen, Caen, France; ^11^ Caen Basse Normandie University, Caen, France

**Keywords:** patient-centered care, quality of life, care, qualitative study, crossed perspectives

## Abstract

**Purpose:**

Patients, family members, and physicians participate in cancer care, but their perspectives about what is helpful during cancer treatment have rarely been compared. The aim of this study was to compare these three perspectives.

**Methods:**

Multicenter qualitative study (with previously published protocol) based on 90 semi-structured interviews. Participants (purposively selected until data saturation) came from three different subsamples: (i) patients with cancer (n=30), (ii) their relatives (n=30), and (iii) their referring physicians (n=10, interviewed more than once).

**Results:**

Our analysis found 3 main axes (perceived positive effects of cancer treatment, perceived negative effects of cancer treatment, doctor-physician relationship), each composed of 2 main themes. The findings showed that patients, families, and physicians shared the long-term objective of increasing survival (while reducing side effects). However, patients and relatives also pointed out the importance of living with cancer each day and thus of factors helping them to live as well as possible in daily life. The physicians difficulty in coping with patients suffering may limit their access to elements that can improve patients capacity to live as well as possible.

**Conclusions:**

During cancer treatment (and not only at the end of life), attention should be given to enhancing the capacity of patients to live as well as possible (not only as long as possible) to meet the goals of patient-centered care and satisfy this important need of patients and families.

## INTRODUCTION

The last decade has been characterized by dramatic progress in oncology from advances in modern medicine, genetics, and the development of evidence-based treatment. At the same time, as the center of medical relationships has shifted from the doctor to the patient, the decision-making process has come to focus on patients’ preferences, choices, and needs, for these influence their therapeutic choices, satisfaction, and quality of life during and after treatment [1–4]. This change has led physicians to move beyond the traditional biomedical model and try to see cancer through their patients’ eyes [5, 6]. The importance of families and their involvement in shared decision-making during cancer treatment is also receiving increased recognition [7].

Nonetheless, patients and families have significant psychosocial needs during the cancer treatment, survival, and end-of life periods that remain unmet [8–13]. Comparisons of the perspectives of patients and family members with those of physicians are rare in oncology, especially when assessed with qualitative methods. A recent review of quantitative and qualitative studies on physician-patient-companion communication and decision-making identified only four qualitative studies on the subject [14]. These studies, however, focused on the perspectives of healthcare professionals [15], of patients and health-care professionals [16], or of patients and family members [17, 18]. The only studies including the perspective of the three protagonists of cancer care for these issues have thus far looked only at specific aspects of cancer care (i.e., the implementation of couple-focused psychosocial care in routine cancer services, or family involvement in treatment decision making) [19, 20]. These findings indicate that further research is needed to deepen our understanding of the perspectives of patients, family members, and physicians during cancer care.

This study used qualitative methods with the aim of exploring: (i) patients’ perspectives about their treatment (what helped them during their treatment in terms of both care and cure, what enabled them to handle their situation better, and what made their illness harder for them), (ii) their families’ perspectives about what was helpful during the treatment, and (iii) their physicians’ perspectives of what helped the patients. We compared these three points of view to shed light on the convergences and divergences in these perspectives.

## MATERIALS AND METHODS

This national multicenter study involved oncology departments at three university hospitals, two located in the Paris area (Saint-Louis Hospital, Paris, and Avicenne Hospital, Bobigny), and one in northern France (Caen). The Paris-Descartes University review board (CERES) reviewed the protocol (previously published [21]) and approved it (IRB number: 20140600001072). All participants provided written informed consent.

### Participants and recruitment

Recruitment followed a purposive sampling strategy with maximum variation. This strategy allowed us to include a variety of cases that differed with respect to cancer site and type, stage, duration of treatment, age, and relationship of family member. Unlike other recruitment strategies in qualitative research (i.e., homogeneous or convenience sampling), this strategy aims to “challenge” the findings continuously by including participants who might invalidate what was previously found and thus lead to a broader understanding of the phenomenon under study [22]. Inclusion of participants continued until saturation was reached (i.e., until subsequent interviews provided no new elements). Saturation is a key validity criterion in qualitative research, as it ensures that the phenomenon is being explored in-depth and that further interviews are unlikely to produce new findings [23]. Three subgroups of participants were interviewed (inclusion criteria are reported in Table 1): (i) patients with cancer (*n* = 30), (ii) one relative for each patient (*n* = 30), and (iii) the patients’ referring physicians (*n* = 10) (Table 2). We conducted 90 interviews. Physicians were interviewed more than once (on average, each physician participated in 3 interviews, range 1-6). We began by discussing the inclusion criteria with the physicians who agreed to participate during pre-study meetings and asking them to identify 3 to 5 suitable patients who met the inclusion criteria. These patients were chosen in line with the purposive sample strategy and the saturation criterion, and subsequently contacted by their treating physician. During this first contact, the physicians asked the patients to participate in the study as well as whether they had a family member who would agree to participate as well. Patients were free to ask any family member they chose. The physicians briefly explained the study objectives during this first contact, and the researchers re-explained it to them in detail during their meetings with each participant (when the informed consent form was signed). The patients’ mean age was 63 years (range 28-91), and 57% were women. In most cases (25), the family member (chosen by the patient) was the spouse/partner. Others were mothers (2), daughters (2), and a grandmother (1). Oncologists (mean age 45 years; 50% women) worked in either medical oncology or dermatology departments.

**Table 1:** Inclusion and exclusion criteria

Inclusion criteria	Exclusion criteria
· Age: 18 years or older (no upper limit) · Treatment started at least 6 months before the interview · A close relative agrees to participate in the research · Referring physician agrees to participate in the research · Able to communicate in French	· Age: <18 years · In the terminal phase (expected survival less than 6 months) · No relative or referring physician willing to participate in the research.

**Table 2:** Summary of participant characteristics

		n (%)
Patients	Gender, women	17 (57)
(n=30)	Age, mean y	63.5
	Cancer type	
	Breast carcinoma	9 (30)
	Lung carcinoma	1 (3)
	Melanoma	7 (23)
	Skin lymphoma	6 (20)
	Bladder/kidney carcinoma	3 (10)
	Prostate carcinoma	1 (3)
	Testis germ-cell cancer	1 (3)
	Ovaries	2 (7)
	Disease stage	
	Metastatic	14 (47)
	Localized	16 (53)
	Treatment received	
	Intravenous chemotherapy only	6 (20)
	Intravenous chemotherapy + other(s)	19 (63)
	Oral chemotherapy, other treatments	5 (17)
	Duration of cancer treatment period	
	Less than 1 year	6 (20)
	1 to 3 years	6 (20)
	3 to 5 years	12 (40)
	More than 5 years	6 (20)
	Hospital	
	Paris, Saint Louis Hospital	15 (50)
	Bobigny, Avicenne Hospital	3 (10)
	Caen, University Hospital Centre	12 (40)
Family	Gender, women	15 (50)
(n=30)	Age, mean y	64
	Member	
	Spouse/Partner	25 (83)
	Daughter	2 (7)
	Mother	2 (7)
	Grandmother	1 (3)
Physicians	Gender, women	5 (50)
(n=10)	Department	
	Medical oncology	5 (50)
	Dermatologic oncology	5 (50)
	Age, mean y	45
	Experience, mean y	27

To ensure methodological rigor, we adopted a maximum variation purposive sampling strategy: we focused on a variety of cancer treatment experiences (related to various types of cancer, which differ in their prevalence, mortality rate, and clinical manifestations), both genders, and different teaching hospitals.

### Data collection

Experienced qualitative researchers (MO, JS, and ML) conducted semistructured interviews according to a topic guide of open questions (Table 3) intended to explore the treatment experience from the perspective of these 3 groups. We focused especially on the perceived positive and deleterious effects of treatment and on the patient-physician relationship. Participants were interviewed in a private room in the hospital of treatment; interviews were audiotaped and transcribed verbatim.

**Table 3:** Interview topic guide

**Topic 1: Story of the illness**
**Topic 2: Focus on the care received** Allopathic treatments (chemotherapy, radiotherapy, surgery) Complementary treatments (alternative/complementary medicines, psychosocial treatment, self-help group) Relationship with doctors **Topic 3: Coping with the emotional burden**

Note: for each topic, interviewers focused on what was helpful/positive, and what was deleterious/negative

### Data analysis

We performed a thematic content analysis. First, we coded the material with descriptive codes. Then conceptual notes were drafted, by condensation, comparison, and abstraction of the initial notes. Connections with notes were then mapped and synthesized, and emergent themes developed. The interviews of each individual trio (patient, family member, and physician) were analyzed together, to compare and contrast their perspectives.

Three researchers (MO, JS, ARL) independently analyzed all interviews, using Nvivo 10 software. After negotiation of disagreements and discrepancies within the research team (QualiPRO study team) during regular meetings, consensus was reached on all findings. The study is reported according to the COREQ statement [24].

## RESULTS

Our analysis found 3 main axes, each composed of 2 main themes. The results are presented below, with references (in brackets) to supporting quotations from the interviews (in Table 4).

**Table 4:** Quotes exemplifying the thematic results

*Q1.* All the treatments we've given him have helped in the sense that they were able to regain control of the disease, to keep it from getting worse, at least temporarily […] with hormone-dependent cancers such as breast cancer, we can, with medical treatments such as radiation therapy or hormone therapy prevent or at least slow down the advance of the disease and so give the patient uh a hope of survival uh of several years without being able to be more precise and with a quality of life that we hope will be the least poor possible (Physician).
*Q2.* It's very effective, that is, he's in complete remission. The scanner no longer shows anything (Physician).
*Q3.* Well, no because there is no problem. It's with the exams that we see it. When he does the chemo, there are also the blood counts and all that. And then once the PSA is really low, we stop. When the PSA goes down, that gives us a little hope (Family member).
*Q4.* If she weren't there. I don't think that I'd be here. She's done everything, absolutely everything. She does everything for me. […] when she talks, I'm cured. Almost (Patient).
*Q5.* After you have to adapt to each phase. Her reaction, her behavior about what the patient is experiencing. To try to understand, support, sometimes hide your own fears because... you don't have the right to add … well, I don’t think that you have the right to add on to the patient's worries. On the contrary, you have to keep the energy level up (Family member).
*Q6.* You take it on yourself. You can't let him see that you have no hope. You're the only person he has in the world. If you show little signs of weakness, he's not going to do well. So you take it on yourself. … What I say is: one cancer = two deaths. … …And I don't think I'm wrong (Family member).
*Q7.* It was the hardest moment for me because I was so weak, well, I hid everything, I didn't want to inflict it on my husband so, I'm telling you, I would go upstairs to the bedroom and I... I did absolutely nothing, I sat in an armchair and as soon as I heard someone coming upstairs, I reacted so that ... they wouldn't see, because I didn't want to make everyone miserable (Patient).
*Q8.* One day I had a chemo treatment that was hard. I could hardly walk. So Friday morning I said to my wife, let's load the truck and I'll go at 2 this afternoon, I'm going to S. to set up the bingo. Good, she says to me. She is brave. Ok. It takes some time to load all that. There's a whole list. It doesn’t look like much but you need quite a lot. I started to load it but I couldn't. I tried but I couldn't. She told me to go lie down, she'd do it. I went to lie down. I woke up at a quarter to four. She had loaded the truck, and she helped me to climb in. The neighbor across the street said to us, don't go, you're going to kill yourself, he's not capable of driving. I said, yes, yes, yes, yes. I'm sitting in my truck, good. Exit the courtyard, perfectly straight, first, second, indicator, turn right, 5^th^ gear then 6th. After 25 km I'm 80% ok (Patient).
*Q9.* [Talking about his wife with cancer] physical therapy or yoga, to maintain flexibility, mobility and a sense of mastery over the physical body. So inside you’ve lost control…but outside you feel control of your body (Family) that helped her so much to be able, even, just to move her arm and since the PT (physical therapist) was nice, so she said really good things about it [..] he laughed with her, told her jokes, and so uh, he gave her confidence (Family member).
*Q10.* I don't know if her family… her family is a little blurred. I've seen her come with her partner, who is kind of mysterious. I don't know if he's got a very important place in her life. I learned today that he lives in Bordeaux while she lives in Paris. Well you know… I must have seen him twice. I don't think he was here the first time. I don't remember. He is a little transparent (Physician).
*Q11.* The first chemo was very good, the second uh a little less good , the third still less good and the fourth was truly awful […] he was so sick that he said, I think I've had it! (Family member).
*Q12.* The treatment, it cuts both ways, it doesn't do her any good in the sense that she could... she wasn't eating much, she wasn't moving much, but it helped in the sense that she needed the treatment to get better (Family).
*Q13.* [speaking about her job as social worker] How do you want me to counsel anyone now, I'm not myself, I can't anymore, I'm afraid I'm going to crack up. You have to stay calm (...) Now I'm incapable of that, I fall apart or I get crazy angry (...) and I don't have the right to do that (Patient).
*Q14.* It's horrible because you're not the same, you don't have the same desires, or the same priorities and the other person, who you live with, doesn't understand you anymore (Patient)
*Q15.* The most ... what you felt the most was the chemo. Because chemo, it’s the physical, destructive effect, physical destruction causing a mental shock. So that was the hardest (Family member).
*Q16.* She said to Dr M, she said to him, well, I feel that things are going well, and I’m telling you that on July 6, I’m going to Avignon and I am going to stay there a month. Deal with it. I am totally confident. Do your job as a doctor and I, I will do everything you tell me to do, but I’m going to Avignon. […] and Dr M said, “Oh, ok,” because he knew that with her, if he said no, she was going to be depressed, he would be eliminating what’s the most important thing for her all year, that’s Avignon [Avignon’s theatre festival]… Dr M knows that and that’s why he is so great, because he never kept her from doing something that she said, ‘I want to do it.’ (Family member).
*Q17.* And then, now, he [the doctor] is with me, he will stay with me. He will support me, I'm sure. After a while, you get the idea that there is nothing to do because, in fact, my cancer, it's not getting better! (Patient).
*Q18.* There's a kind of intimacy or in any case, much deeper confidence that has developed since then because she was always saying, "I want to get out of this, I want to get better, saying all the time that she knows she won't be cured, but that she wants to pull through (Physician)
*Q19.* She was pretty exceptional, in any case, I found her very courageous, she's a fighter, and she’s someone who's fairly cheerful. Finally I find her very pleasant. Good. There are plenty of things that make a patient pleasant, I find. (Physician).
*Q20.* I was really in bad shape and so tired besides that I said to myself that I wasn't well ... um, I had really, you can't call it depression but I really was not well. Interviewer: The blues Yeah, really but major, because really I didn’t feel like getting up, I didn't want anything, the telephone it was, I just didn’t want it, it was too hard, everything was complicated, hard to do, nothing was going right and I didn't want anything (Patient). This patient profile and I realize now, it's also those who I ask perhaps not so much because they give the impression they're doing ok and finally um there it is, I wonder less about their family, about a solitude that exists perhaps but she uh doesn't seem to be suffering from it anyway (her physician).
*Q21.* The people I treat, that I see, um, there's a framework, a profile, there are people I can't treat, for example, people who are too passive, who aren't active in their disease, here, these are people who are perfectly active; you say something to them, they think about it, they say yes or they say no, they think about it, I mean, they’re not like I come here, and I just wait for it to be over, they’re not like that (Physician).

Note: This table reports exemplary quotations supporting our results. Q1 to Q21 are referred to in square brackets in the results section.

### Perceived positive effects of cancer treatment

#### Survival as treatment efficacy

Patients, family, and physicians all judged the effectiveness of cancer treatment (e.g., chemotherapy, radiotherapy) by its postponement of death or its prevention or by a cure, when possible. Thus everyone considered survival the primary aim of treatment [Q1]. Physicians especially focused on objective response criteria for treatment efficacy, i.e., clinical, radiological, or laboratory data, such as prostate-specific antigen levels in prostate cancer. The physician's explanations made these data the markers of treatment efficacy for the patients and their families as well [Q2-Q3].

#### Ability to live as well as possible

Although survival was the main long-term objective for physicians, families, and patients (who were willing to accept burdensome side effects to increase their chance to survive), patients and families also insisted on the need to cope with everyday life. When interviewed about what was helpful to them during the treatment period, patients and family mentioned two main factors that helped them to live daily life as well as possible: (i) the presence of family members or another emotional bond, and (ii) involvement in a meaningful activity.

Most patients described the presence and everyday support of the family as extremely helpful in coping with their illness. Family members echoed this perspective, underlining their adaptation to make the patients’ everyday lives easier, to support them during the hardest moments, and to share their burden [Q4-Q5]. This helpfulness was difficult and psychologically grueling for these family members [Q6], as patients recognized. In turn, some patients felt they had to help support their relatives, even to the extent of hiding their physical and emotional feelings [Q7].

Patients described involvement in different kinds of meaningful activities. These shared some characteristics: (i) they were a part of life uncontaminated by issues related to illness or treatment, (ii) they were shared with the partner/family, (iii) they fostered hope and thus showed the possibility of life beyond cancer. These activities helped the patients cope with their illness and gave them something to do beyond their medical routines [Q8]. Other activities included supportive care (e.g., physiotherapy), complementary practices (e.g., yoga), and cancer support groups [Q9].

Physicians knew in general that both the presence of family members and meaningful activity are helpful. They were sometimes unaware, however, of the actual presence or role of either the family or the activity, and they very seldom used this information as a therapeutic lever (for example, to motivate and encourage patients to pursue or to help them to find a meaningful activity) [Q10].

### Perceived deleterious effects of cancer treatment

#### Side effects

Patients, family, and physicians agreed that the side effects of cancer treatment were deleterious. Patients clearly described the treatment as destroying the body: the further they progressed toward completion of treatment, the worse they felt [Q11]. Sometimes, these side effects were the only experience of the treatment, with no perception of improvement. This negative experience means that patients cannot physically perceive the efficacy of treatment in reducing disease but are required to believe in and trust the doctor and the drug. Families had the same view about the side effects of these treatments [Q12].

Physicians grasped the burden of chemotherapy side effects and understood that coping with these is the principal challenge for patients during the treatment period.

#### Impact of side effects on the ability to live well

Beside their direct physical effects, side effects strongly influenced patients’ lifestyle, work, body image, and ability to make plans. All patients reported, to different degrees, a sense that their identity was undermined. This erosion of self affected their capacity to live in the world, to work, and to carry on with ordinary family life [Q13]. Family members too were concerned by the vulnerability induced by the cancer and its treatment [Q14-Q15].

Physicians could, on the whole, assess the direct physical impact of side effects. In contrast, they found it more difficult to understand their psychological impact. It is unclear, however, if this discrepancy resulted from physicians’ difficulties in discussing psychological or emotional topics, or from the patients’ and families’ perception that these are not appropriate topics to discuss with their oncologists. In either case, sharing this aspect within the medical framework was difficult.

### The patient-physician relationship

Beyond medical treatment and care, the patient-physician relationship appeared important to both protagonists. Within this relationship, each characterized the other symmetrically.

#### Perspective of patients and families about the physician and the patient-physician relationship

According to patients and their family members, good physicians deal with non-medical as well as medical issues, providing care that extends beyond management of cancer and the side effects of treatment. They described these doctors as caring about their patients’ ability to live their lives as normally, as well, as possible and provided the care required to make that possible - above and beyond care to prevent, avoid, or delay death [Q16]. The physicians’ role in improving quality of life takes different forms depending on the treatment period but remains in evidence even in the final stages (when care is exclusively palliative). It always assumes a commitment to an authentic relationship [Q17].

#### Perspective of physician about the patient and the patient-physician relationship

Physicians described the patients with whom they felt most comfortable, independently of clinical characteristics, that is, based on patients’ personal dispositions.

Regardless of each patient's individuality, we observed a continuum in physicians’ descriptions of their patients’ traits, and its opposite ends had different consequences for clinical practice. At one extreme were the patients who faced their illness positively, with optimism, and with few negative emotions, who could mobilize their own resources to cope with their cancer and the negative aspects (somatic and emotional) of its treatment. The terms trust, intimacy, and “good patients” were inevitably intertwined [Q18]. These patients and their attitudes induced greater physician involvement and intimacy in the relationship [Q19], which in turn improved trust. Nonetheless, this attitude kept oncologists from exploring in depth how patients experienced their illness outside the medical setting and beyond what they showed in consultation and thus sometimes led the doctors to overlook patients’ real feelings [Q20]. At the other extreme are the patients with whom doctors found it difficult to work. These were the patients unable to see anything positively during their illness, who could not take comfort or even pleasure in little steps forward, and who were demanding. Physicians found it difficult to provide support for these patients and sometime misinterpreted their attitudes, intentions, and feelings [Q21].

## DISCUSSION

Our analysis of the perspectives of patients, their relatives, and their physicians toward cancer treatment showed that all participants shared the long-term objective of increasing survival (while reducing side effects). Nonetheless, it was mainly the patients and their family members who also pointed out the importance of living with cancer on a day-to-day basis and thus of the elements that enable them to live as well as possible in their everyday activities.

The presence of family members (loved ones) and involvement in meaningful activity emerged as the main elements of a good quality of life. Although these points were previously known, most physicians have not yet fully understood them, and their use as therapeutic tools has been relatively sparse. Thus, these factors optimizing patients’ ability to live the best lives possible have remained marginal and could not become a focus of cancer treatment. These findings echo those of other studies about the needs — met and unmet — of patients treated for cancer and their family members. For example, studies have showed that physicians fail to take into account the spiritual and religious concerns of their severely ill patients [25–28], despite recommendations [29], patients’ preferences [26], and the demonstrated benefit in terms of patients’ satisfaction [27]. These reports are evidence of mismatched perceptions between patients and doctors [30]. Similarly, families play an important role in fostering the patients’ ability to live as well as possible in all phases of the cancer journey (diagnosis, treatment, palliative, and end-of-life periods), as one study has demonstrated [31]. Nevertheless, for families to fulfill this critical function, they require support that meets their psychological, spiritual, physical and social needs [32].

Why do physicians have such limited access to the factors helping patients to live their daily lives as well as possible? Our third axis (the patient-physician relationship) suggests that oncologists’ capacity to embrace patients’ negative feelings and emotions could be a barrier to their access to the factors that improve the patients’ capacity to live as well as possible. Our qualitative analyses revealed that when patients explicitly expressed their suffering and struggle to cope with their disease-related negative emotions, their physicians found it difficult to deal with these emotions during consultation. This has also been shown in other settings, for instance, in relation to physicians’ lack of training in discussing spiritual concerns [32,33]. On the other hand, when patients who appear able to cope effectively with their illness do not unambiguously articulate these negative emotions, physicians find it difficult to go beyond this superficial image and inquire about the patients’ hardships. This distance may be due to the attitudes of patients who prefer not to disclose their emotion and want to focus only on medical subjects, but also to physicians who lack the tools to gain access to and deal helpfully with patients’ emotions).

In either case, this distance creates the risk of overlooking patients’ suffering and failing to mobilize the resources (family members or meaningful activities or both) that will optimize their quality of life during the treatment period.

The increased survival of cancer patients due to advances in oncology has led to longer, chronic disease and requires them to be in treatment for longer periods (e.g., multiple courses of chemotherapy, alternating radiotherapy and chemotherapy, side effects, and pain). Two consequences are worth noting. First, prolonging treatments and only focusing on the *quantity* of life (sometimes with overaggressive treatment) [34,35] does not fit all patients’ needs, and in some cases (e.g., end-stage cancers) may not improve survival [36]. Nor does it meet the needs of family members, who do not experience either better bereavement [36] or relief from their stress, pain, and suffering [37]. The overwhelming limitation of these issues to the field of palliative medicine [38] and the short shrift given them in curative medicine creates an illusion that these topics are important only when death can no longer be postponed (i.e., when no curative treatment exists) [39]. Any cancer, however, is an arduous experience, regardless of its prognosis and the extent of the treatment burden. Our study shows that physicians are interested in the future (survival) but fail to pay enough attention to the present by fostering the capacity to live as well as possible during the daily life of treatment. The psychosocial needs reported in cancer survivors [11] and their families [40] exemplify the extent to which these issues have been neglected during the treatment phase.

Second, with the increased chronicity of cancer comes the need for a different kind of care relationship, one in which physicians can cope with — and help patients to cope with — the patients’ needs and feelings (including negative emotions) beyond medical management and can assume the relational dimension of care. Although professional psychological interventions are useful for patients facing severe illness [41], not every patient needs these interventions. Splitting care and cure (and outsourcing care to other professionals) can produce silo cultures and negatively affect patient management and satisfaction [39]. Improving the education of oncologists may be a key factor in preventing this gap between care and cure, by preparing physicians to assume this dimension of care and develop awareness of their own practices. One viable educational intervention, for instance, could be the implementation of discussion groups (such as Balint groups, designed to improve clinicians’ skills by discussing personal cases focusing on patient-doctor interaction) [42]. The effectiveness of these groups has already been shown in several areas, including improving communication skills, dealing more effectively with patient needs, and managing physicians’ involvement in their patient-physician relationships; it also increases physicians’ well-being [43–45].

Finally, improved knowledge about cancer support groups and complementary medicine could enable physicians to refer patients to these activities, which appear to meet some of their needs.

## STRENGTHS AND LIMITATIONS

Our qualitative study was multicenter and rigorously conducted, with a sample size (*n* = 70, 90 interviews, which is larger than usual in qualitative studies) and variety of cancer types that allowed us to explore different situations. These factors enhance the transferability of our findings to other cancer contexts. However, as cancer treatment varies according to the medical system, the first limitation concerns the setting of the study. Hence, transferability to other medical systems, especially those in non-Western countries, requires caution. A second limitation is the fact that physicians are interviewed multiple times (one interview to discuss each patient), which may have over-emphasized the individual relationship style of some of them. Finally, our recruitment process might have limited our findings. Although all the patients we contacted found a relative who agreed to take part in the study, the doctors were unlikely to suggest patients without any social network for recruitment, and our findings cannot be generalized to them.

## CONCLUSIONS

Patient-centered cancer treatment requires that physicians integrate the dimension of care into the curative treatments performed. As patients certainly agreed, their survival should be a priority for physicians, but equal attention should be given to enhancing their capacity to live as well and not simply as long as possible. The elements needed to meet this aim exist and can be accessed within the patient-physician relationship. Specific medical education has the potential to increase physicians’ ability to recognize, elicit, and use these elements as a therapeutic tool and to cope appropriately with patients’ negative emotions. In difficult life-threatening conditions, the role of the physician might be summarized by the adage of Hippocrates: “To cure sometimes, to relieve often, to comfort always”.
